# An engineered probiotic secreting Sj16 ameliorates colitis via Ruminococcaceae/butyrate/retinoic acid axis

**DOI:** 10.1002/btm2.10219

**Published:** 2021-04-02

**Authors:** Lifu Wang, Yao Liao, Ruibing Yang, Zifeng Zhu, Lichao Zhang, Zhongdao Wu, Xi Sun

**Affiliations:** ^1^ Department of Parasitology, Zhongshan School of Medicine Sun Yat‐sen University Guangzhou China; ^2^ Key Laboratory of Tropical Disease Control Ministry of Education, Sun Yat‐sen University Guangzhou China; ^3^ Provincial Engineering Technology Research Center for Biological Vector Control Guangzhou China

**Keywords:** colitis, engineered probiotic, Ruminococcaceae/butyrate/retinoic acid axis, Sj16, Treg/Th17 balance

## Abstract

Most inflammatory bowel disease (IBD) patients are unable to maintain a lifelong remission. Developing a novel therapeutic strategy is urgently needed. In this study, we adopt a new strategy to attenuate colitis using the *Escherichia coli* Nissle 1917 probiotic strain to express a *schistosome* immunoregulatory protein (Sj16) in the gastrointestinal tract. The genetically engineered Nissle 1917 (EcN‐Sj16) highly expressed Sj16 in the gastrointestinal tracts of dextran sulfate sodium‐induced colitis mice and significantly attenuated the clinical activity of colitis mice. Mechanistically, EcN‐Sj16 increased the intestinal microbiota diversity and selectively promoted the growth of Ruminococcaceae and therefore enhanced the butyrate production. Butyrate induced the expression of retinoic acid, which further attenuated the clinical activity of colitis mice by increasing Treg cells and decreasing Th17. Strikingly, retinoic acid inhibitor inhibited the therapeutic effects of EcN‐Sj16 in colitis mice. These findings suggest that EcN‐Sj16 represents a novel engineered probiotic that may be used to treat IBD.

## 1 INTRODUCTION

Inflammatory bowel disease (IBD), which includes Crohn's disease (CD) and ulcerative colitis (UC), is a chronic intermittent disorder characterized by epithelial barrier damage and intestinal inflammation.^1^, ^2^ Although the exact etiologies underlying IBD development remain unclear, its pathogenesis is associated with multiple factors, including genetic susceptibility, immune activity, environmental changes, and qualitative and quantitative gut microbiota abnormalities.^3^, ^4^ IBD prevalence is estimated to exceed 0.3% in many developed countries, and the incidence is rising in newly industrialized countries.^5^

Parasitic worms have evolved to modulate host responses that enable the worms to survive in immunocompetent hosts by inducing an anti‐inflammatory environment.^6^ Parasite‐derived immunoregulator molecules have been exploited to ameliorate IBD in both mice and humans. A clinical study demonstrated that *Trichuris trichiurids* has shown considerable promise for treating patients with UC.^7^ Studies involving rodents have indicated that the hookworms and *Trichinella spiralis* exert protective effects against colitis.^8^, ^9^ Additionally, *Schistosoma japonicum* and *Schistosoma mansoni* can attenuate colitis in mice.^10^, ^11^ However, therapies using live helminths present safety issues, and researchers have shifted their focus to helminth‐derived molecules. Our group initially described Sj16, a 16‐kDa protein secreted by *Schistosoma japonicum*. Our previous studies revealed that Sj16 has immunoregulatory effects and exerts protective effects against dextran sulfate sodium (DSS)‐induced colitis.^12^

Most IBD patients cannot maintain lifelong remission and therefore must tolerate symptomatic relapse and need long‐term medication. Thus, novel therapeutic strategies that can replace long‐term medications are urgently needed. We proposed to genetically engineer naturally occurring bacteria that secrete Sj16 to consistently protect against colitis. Introducing such genetically engineered bacteria that can stay in the mucosa and become stable components of the resident microbiota of IBD patients would provide a potential long‐lasting treatment. In this study, we used the commensal strain, *Escherichia coli* (*E. coli*) Nissle 1917 (EcN), as a carrier. EcN produces no known virulence factors, is safe for humans, and is widely used as a probiotic treatment for diarrhea and IBD.^13^, ^14^ Studies have shown that EcN can suppress pathogenic bacterial adhesion and invasion and promote intestinal homeostasis.^15^, ^16^ To secrete Sj16, we used and improved the *E. coli* α‐hemolysin (HlyA) secretion system. HlyA secretion system allows directly exporting proteins from the bacterial cytoplasm to the extracellular environment, bypassing the periplasmic space.^17^

Here, we tested an improved strategy for delivering effector molecules by engineering a probiotic *E. coli* (EcN) strain to secrete Sj16 (EcN‐Sj16) in the gastrointestinal tract. Our data demonstrated that EcN‐Sj16 can live in the gastrointestinal tracts of DSS‐induced colitis mice and significantly attenuated colitis clinical activity via Ruminococcaceae/butyrate/retinoic acid axis.

## 2 RESULTS

### 2.1 EcN‐Sj16 sufficiently secreted Sj16

**FIGURE 1:**
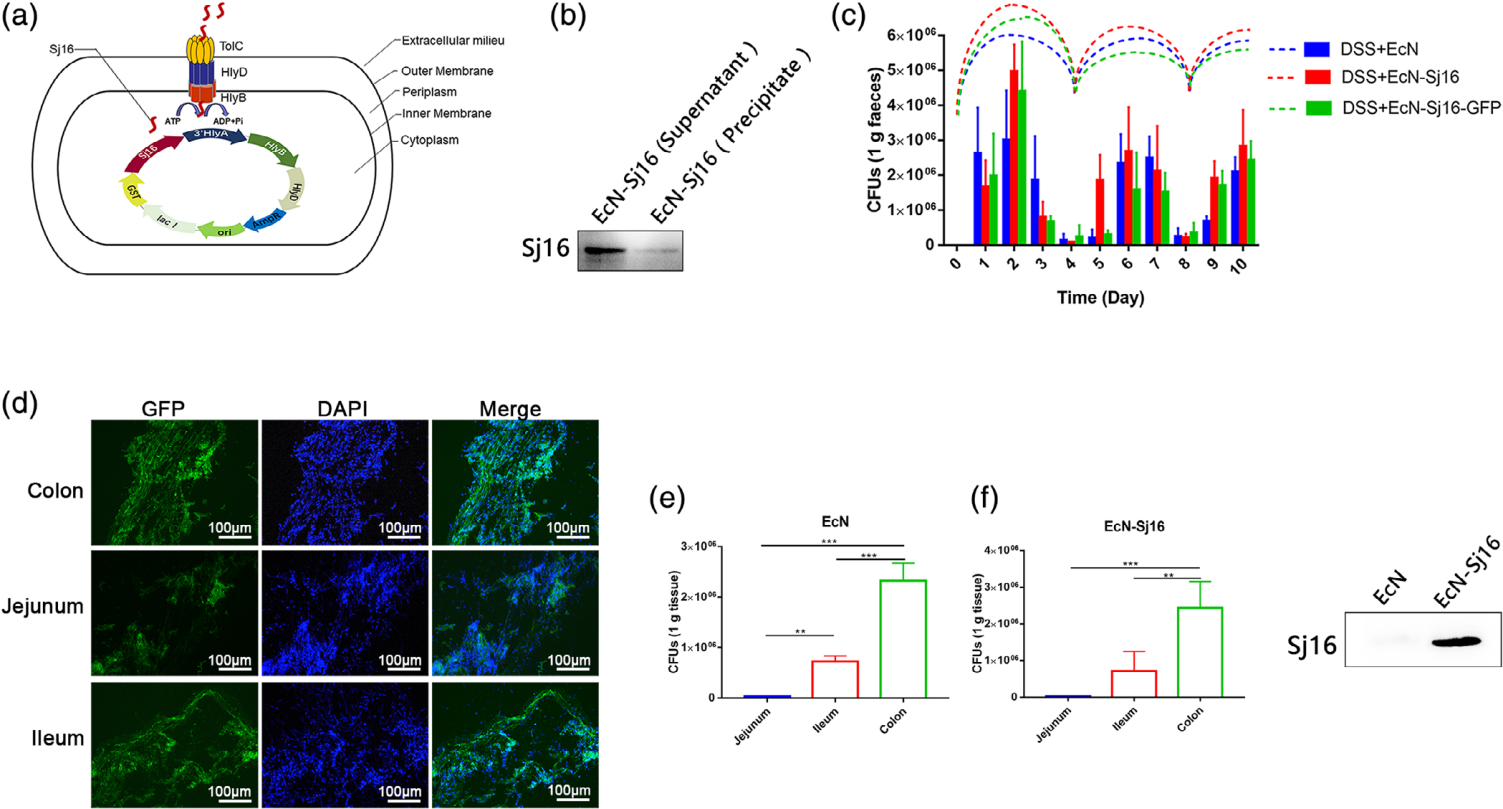
Engineered nissle 1917 (EcN‐Sj16) efficiently secreted Sj16 and colonization in the gastrointestinal tracts of DSS‐induced colitis mice. (a) Schematic representation of the EcN‐Sj16 constructs. (b) Sj16 expressed in the supernatant and precipitate was analyzed by western blotting. (c) Mice were treated with EcN, EcN‐Sj16, and EcN‐Sj16‐GFP on Days 0, 4, and 8. Fecal EcN, EcN‐Sj16, and EcN‐Sj16‐GFP were determined daily using selective ampicillin‐containing plates. The dashed lines show the trends of EcN, EcN‐Sj16, and EcN‐Sj16‐GFP in the feces at each time point. (d) Frozen sections were prepared, EcN‐Sj16‐GFP distributions in the jejunum, ileum, and colon were detected by detecting the content of GFP protein under fluorescence microscope. (e) On Day 10, the mice were sacrificed, and the recombinant bacterial distributions in the jejunum, ileum, and colon were determined. (f) On the fourth day after treatment with EcN‐Sj16, the feces were collected and soaked in PBS and then swirled. The supernatant was extracted by centrifugation at 12,000 g. Sj16 in supernatant was analyzed by western blotting. *n* = 5 per group. Statistical analysis was performed via one‐way ANOVA. **p < 0.01, ***p < 0.001

Our method used a potent colitis therapeutic protein, an efficient secretory system, and a nonpathogenic probiotic strain. Our previous studies showed that Sj16 protected against DSS‐induced colitis without adverse effects.^12^ Therefore, we used Sj16 as a therapeutic protein to treat colitis. For maximum effectiveness of the treatment, the engineered bacteria must secrete Sj16 and allow diffusion into the IBD patient's gastrointestinal tract. We used the *E. coli* HlyA system, which allows directly exporting proteins from the bacterial cytoplasm into the extracellular medium, without a periplasmic intermediate.^17^ The Hly type I secretion system consists of the inner‐membrane components, HlyB and HlyD, and the outer‐membrane component, TolC. The inner and outer membranes form a membrane pore. The HlyB‐HlyD complex recognizes the C‐terminal portion of HlyA to guide the direct export of HlyA‐fusion proteins from the cytoplasm into the extracellular environment, bypassing the periplasmic space (Figure 1(a)). We used EcN as the host.

Previous studies using the HlyA secretion system to secrete exogenous proteins required two expression vectors: one for an exogenous protein fused with the *HlyA* signal sequence and one for the *HlyB* and *HlyD* genes.^18^ However, this design increased the metabolic burden of the host bacteria and increased the control conditions for maintaining expression of two incompatible plasmids. In this study, the Sj16 peptide‐coding sequence was connected with the *HlyA* C‐terminal signal sequence, and the expression vector (pGEX‐4T‐1) included the *HlyB* and *HlyD* genes. This prevented the need to use two expression vectors. The *HlyB* and *HlyD* genes were expressed simultaneously when the expression vector expressed the Sj16 proteins. The HlyB, HlyD, and TolC proteins synthesized by EcN‐Sj16 form a membrane pore, which recognizes the HlyA protein signal at the C‐terminus of Sj16, and Sj16 is secreted directly to the extracellular environment. The EcN‐Sj16 was identified by PCR (Figure S1(a), Supporting Information) and DNA sequencing. To examine EcN‐Sj16 survival in the gastrointestinal tract, we transformed EcN with an expression vector expressing green fluorescent protein (GFP), HlyA, Sj16, HlyB, and HlyD (Figure S1(b),(c)). EcN‐Sj16 highly expressed the Sj16 protein, which was significantly higher in the supernatant than in the precipitate (Figures 1(b) and S1(d)).

### 2.2 EcN‐Sj16 survival in the gastrointestinal tracts of DSS‐induced colitis mice

We investigated the survival of the EcN‐Sj16 in the gastrointestinal tracts of DSS‐induced colitis mice. EcN, EcN‐Sj16, and EcN‐Sj16‐GFP were orally administered to DSS‐induced colitis mice. Fecal CFUs of EcN, EcN‐Sj16, and EcN‐Sj16‐GFP were determined daily using selective ampicillin‐containing plates. Single administrations of 1 × 10^9^ EcN, EcN‐Sj16, and EcN‐Sj16‐GFP led to sustained survival at levels of 10^6^ colony‐forming units (CFU) per gram of feces for 3 days (Figure 1(c)). Because exogenous human bacteria had difficulty competing with the indigenous intestinal microflora of the DSS‐induced colitis mice, the survival levels fell to 10^5^ CFU/g on Day 4. Therefore, we treated the mice with EcN, EcN‐Sj16, and EcN‐Sj16‐GFP on Days 0, 4, and 8. On Day 10, the mice were sacrificed, and the recombinant bacterial distributions in the jejunum, ileum, and colon were determined. EcN‐Sj16 survives in the jejunum, ileum, and colon (Figure 1(d)). The colon contained the highest bacterial concentrations (EcN: 2.3 × 10^6^ CFU/g; EcN‐Sj16: 2.4 × 10^6^ CFU/g). Fewer bacteria were present in the ileum (EcN: 7.2 × 10^5^ CFU/g; EcN‐Sj16: 7.1 × 10^5^ CFU/g) and jejunum (EcN: 3.0 × 10^4^ CFU/g; EcN‐Sj16: 2.1 × 10^4^ CFU/g) (Figure 1(e)). Furthermore, as shown in Figure 1(f), Sj16 can be detected in the supernatant obtained by soaking feces. Although the efficient survival period in mice was only 4 days, colonization may be more effective in humans because EcN is a human strain rather than rodent strain.

### 2.3 EcN‐Sj16 efficiently attenuated clinical activity in DSS‐induced colitis mice

**FIGURE 2:**
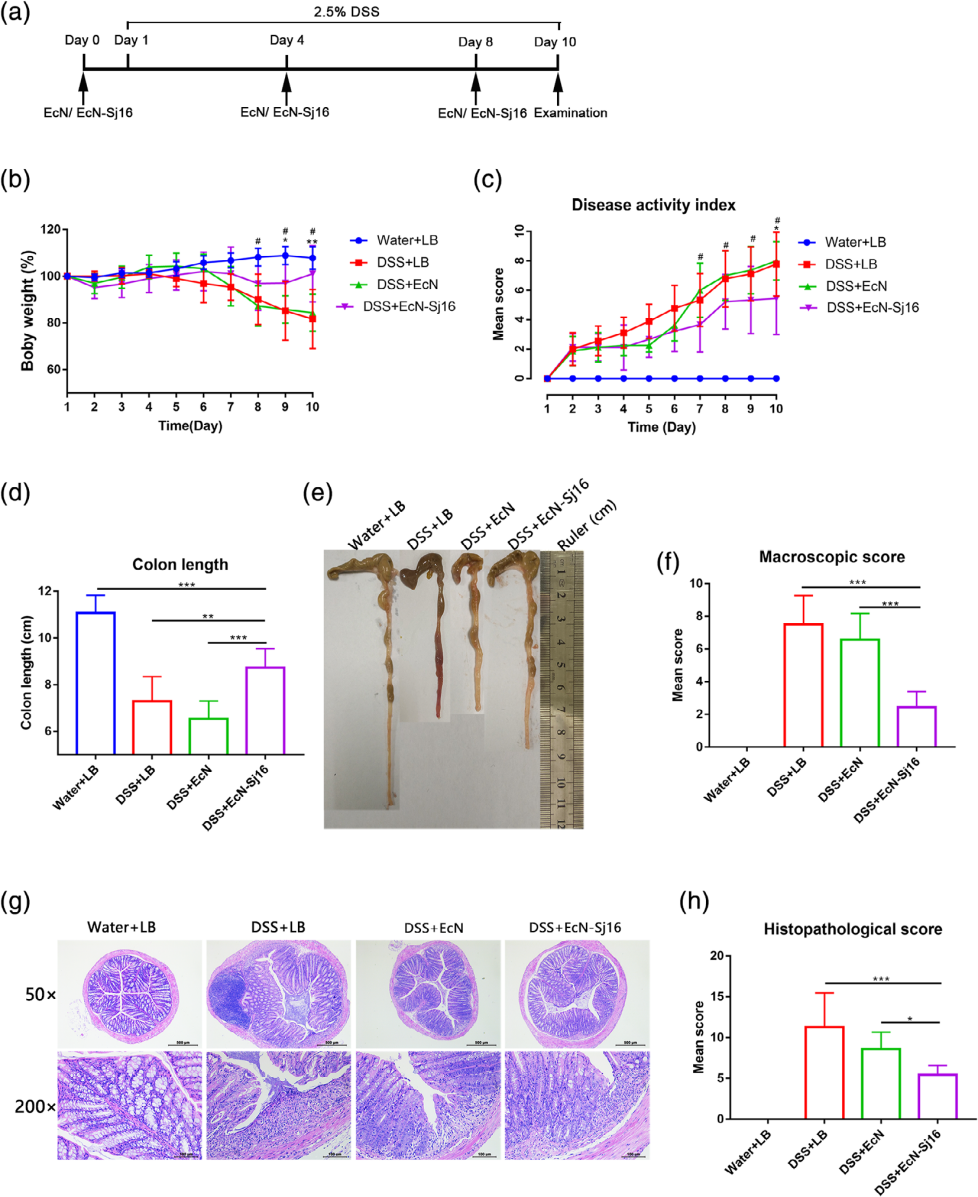
EcN‐Sj16 treatment attenuated clinical activity in DSS‐induced colitis mice. (a) Time schedule of EcN‐Sj16 treatment. (b) Daily mean weight changes per group (*: DSS + EcN‐Sj16 vs. DSS + LB; #: DSS + EcN‐Sj16 vs. DSS + EcN). (c) Changes in disease activity index (DAI) (*: DSS + EcN‐Sj16 vs. DSS + LB; #: DSS + EcN‐Sj16 vs. DSS + EcN). (d) Colon lengths were measured and recorded. (e) Macroscopic appearance of the colons. Mean colon length and typical injury findings are presented. (f) Mean macroscopic scores of the colons. (g) Histopathological changes in the colons were examined via H&E staining (50×, 200×). (h) Histopathological scores of the colons were determined. *n* = 5–8 per group. Statistical analysis was performed via one‐way ANOVA. *p < 0.05, **p < 0.01, ***p < 0.001, # *p* < 0.05

To test whether EcN‐Sj16 attenuated colitis, acute colitis mice were treated with EcN‐Sj16 on Days 0, 4, and 8 (Figure 2(a)). Body weight loss was significantly aggravated in the DSS + LB and DSS + EcN groups from Day 8 after DSS administration. EcN‐Sj16 treatment significantly alleviated the body weight loss in the colitis mice (Figure 2(b)). The disease activity index (DAI) of the EcN‐Sj16‐treated colitis mice was lower than that of the LB‐treated and EcN‐treated colitis mice (Figure 2(c)). DSS significantly reduced the colon lengths in the colitis mice. Therefore, the therapeutic potential of EcN‐Sj16 for suppressing acute colitis was investigated using the colon length. The colon lengths of the EcN‐Sj16‐treated colitis mice were longer than those of the LB‐treated (8.7 ± 1.0 cm vs. 7.2 ± 1.5 cm, *p* < 0.01) and EcN‐treated (8.7 ± 1.0 cm vs. 6.6 ± 1.5 cm, *p* < 0.001) mice (Figure 2(d), (e)). The mean macroscopic colon scores of the EcN‐Sj16‐treated colitis mice were significantly decreased compared with those of the DSS + LB and DSS + EcN groups (Figure 2(f)). Histological assessment showed that DSS significantly disrupted the colonic architecture characterized by significant neutrophil and lymphohistiocyte infiltration, crypt loss, crypt abscess formation, submucosal edema, and goblet cell loss. Treatment with EcN‐Sj16 significantly reduced the colonic architecture disruption compared with that of the LB and EcN treatments (Figure 2(g)). Consistent with these results, the histological scores of the EcN‐Sj16‐treated colitis mice were significantly lower than those of the LB‐and EcN‐treated mice (Figure 2(h)).

### 2.4 EcN‐Sj16 rescued the gut microbial compositions of DSS‐induced colitis mice

**FIGURE 3:**
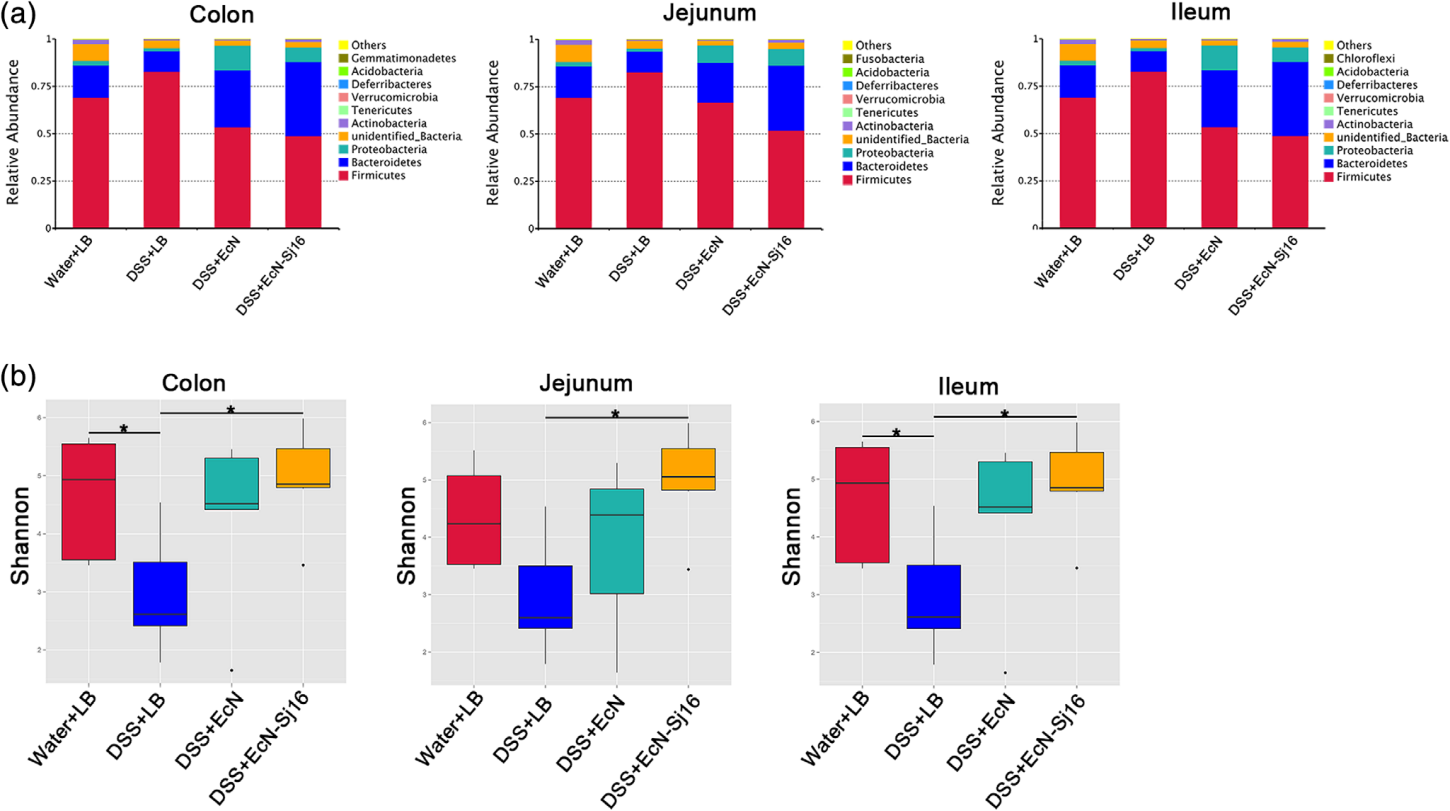
EcN‐Sj16 rescued the gut microbial compositions of DSS‐induced colitis mice. (a) Overview of the microbiota at the phylum level (top 10 species abundance). (b) α‐diversity indices were estimated using the Shannon diversity index. *n* = 3–7 per group. Significant differences in α‐diversity and β‐diversity were assessed via the Wilcoxon rank‐sum test. *p < 0.05, **p < 0.01

Previous studies have shown that the gut microbiota composition is reduced in IBD patients, characterized by reductions in beneficial bacteria and increased pathogens or pathobionts.^19^, ^20^, ^21^ As showed that EcN‐Sj16 survive in the jejunum, ileum and colon (Figure 1(d)), we hypothesized that EcN‐Sj16 would influence the gut microbiota compositions of colitis mice. Using 16S rRNA gene sequencing, we clarified the microbial diversity and compositions of the colon, jejunum, and ileum. An overview of the microbiota at the phylum level showed substantial changes in the DSS + EcN and DSS + EcN‐Sj16 groups compared with those of the DSS + LB group (Figure 3(a)). Operational taxonomic unit (OTU) profiling showed that the microbiotas differed significantly between the DSS + LB and DSS + EcN‐Sj16 groups, whereas the gut microbiotas of the DSS + EcN group were similar to those of the DSS + EcN‐Sj16 group (Figure S2(a)).

To analyze the effect of EcN‐Sj16 treatment on the intestinal microbiotas of colitis mice, we assessed the α‐diversity indices. The Shannon diversity indices and Simpson diversity indices of the colon, jejunum, and ileum were decreased in the colitis mic (Figures 3(b) and S2(b)). After EcN‐Sj16 treatment, these indices were all significantly increased (Figures 3(b) and S2(b)). We next assessed the interindividual dissimilarity using the weighted UniFrac distance‐based β‐diversity index, which considers species abundance between communities. The results revealed decreased microbial β‐diversity indices for the colon, jejunum, and ileum in the colitis mice, which were increased after EcN and EcN‐Sj16 treatment (Figure S2(c)). These results suggest that EcN‐Sj16 rescued the gut microbial compositions of DSS‐induced colitis mice.

### 2.5 EcN‐Sj16 treatment significantly increased the abundances of Ruminococcaceae

**FIGURE 4:**
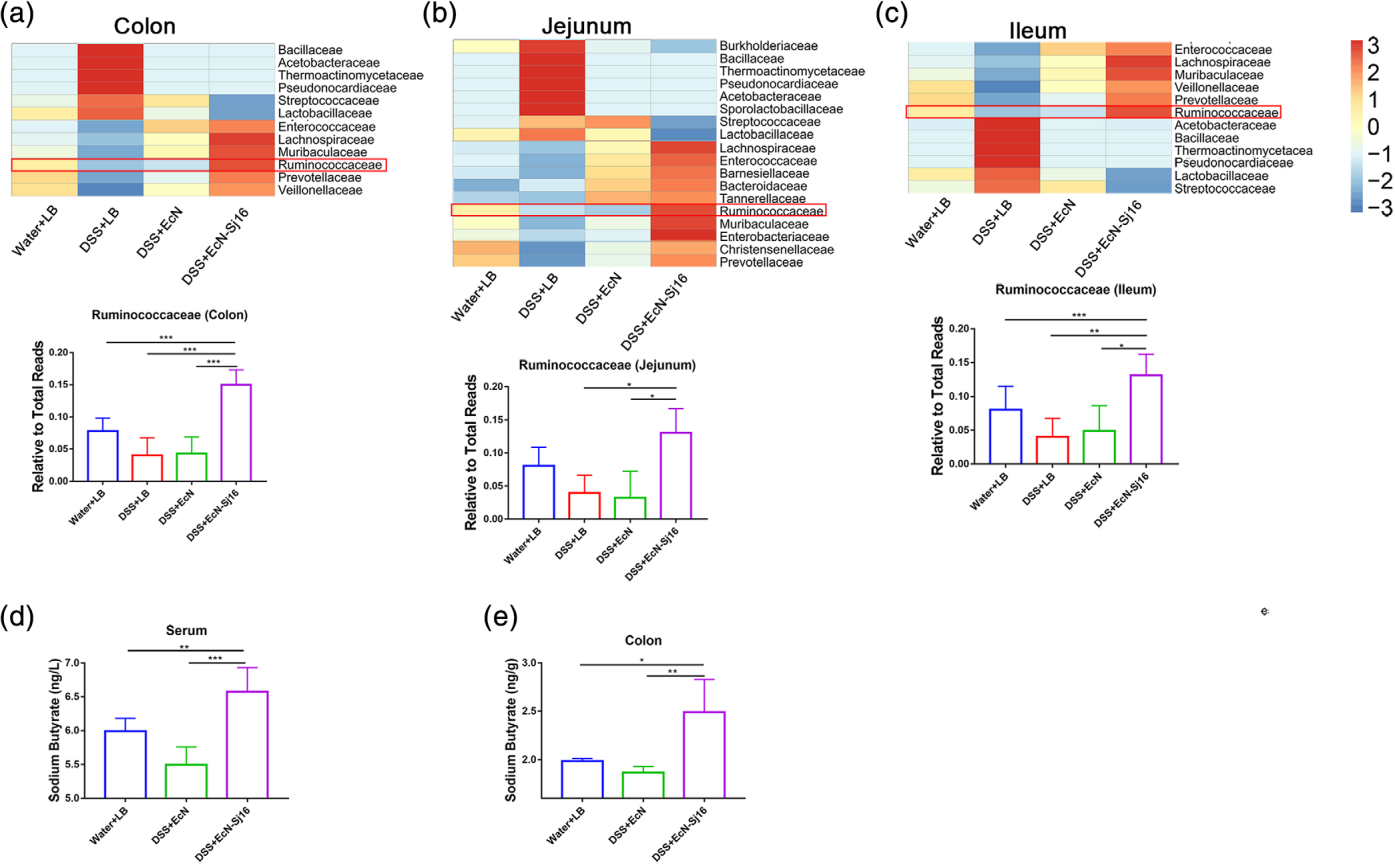
EcN‐Sj16 treatment significantly increased the abundances of Ruminococcaceae. (a–c) Heatmap showing that the Ruminococcaceae family was significantly increased in colon, jejunum, and ileum after EcN‐Sj16 treatment. (d) The level of the sodium butyrate in serum was examined by ELISA. (e) The level of the sodium butyrate in colon tissue was examined by ELISA. *n* = 3–7 per group. Statistical analysis was performed via one‐way ANOVA. *p < 0.05, **p < 0.01, ***p < 0.001

Both EcN and EcN‐Sj16 similarly increased the gut microbiota diversities among DSS‐induced colitis mice. However, EcN‐Sj16 more strongly attenuated the clinical activity of DSS‐induced colitis mice than did EcN, suggesting that some indicator bacterial phylotypes changed after EcN‐Sj16 treatment. We next determined these indicator bacterial phylotypes and asked whether these taxa contributed to the EcN‐Sj16 treatment results. We analyzed the relative abundance of the top10 taxa at the family level and found that Ruminococcaceae family in colon, jejunum, and ileum was decreased in colitis mice, and was increased after EcN‐Sj16 treatment (Figure 4(a)–(c)). In the top10 taxa, Ruminococcaceae was significantly increased in EcN‐Sj16 treatment group than EcN treatment group (Figure 4(a)–(c) and Table S1). Ruminococcaceae was a major butyrate producer and was an important bacteria involved in intestinal health.^22^ Butyrate is an important energy source of intestinal epithelial cells and can promote wound healing.^23^, ^24^, ^25^ We examined the level of the sodium butyrate in serum and colon tissue. As shown in Figure 4(d), (e), sodium butyrate in serum and colon tissue was increased by treating with EcN‐Sj16 compared with EcN treatment.

### 2.6 EcN‐Sj16 increased retinoic acid and regulated Treg/Th17 balance in DSS‐induced colitis mice

**FIGURE 5:**
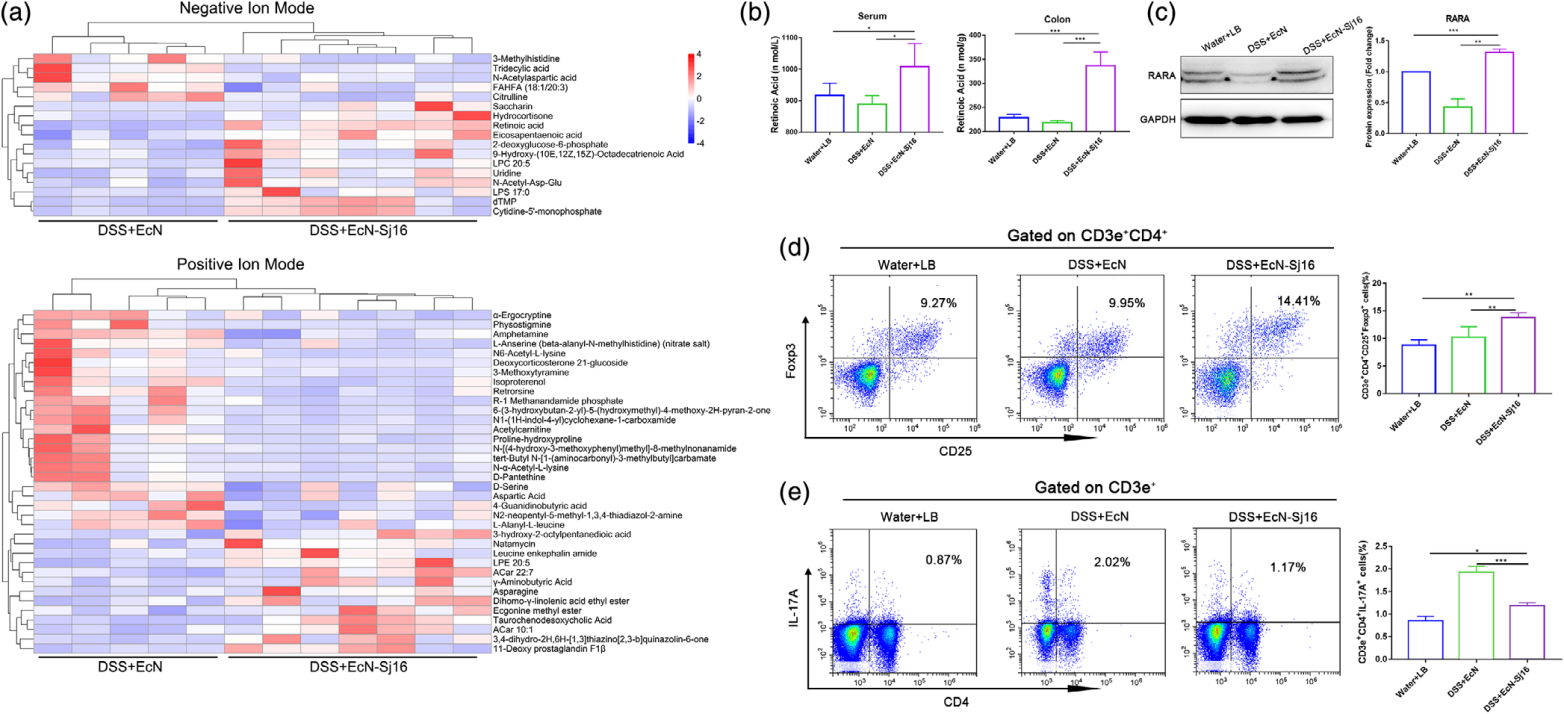
EcN‐Sj16 increased retinoic acid and regulated Treg/Th17 balance in DSS‐induced colitis mice. (a) Metabolite profiles shown as heatmaps in DSS + EcN group and DSS + EcN‐Sj16 group. (b) The levels of the retinoic acid in serum and colon tissue were examined by ELISA. (c) Retinoic acid receptor α (RARA) was analyzed by western blotting. (d) Treg (CD3e^+^CD4^+^CD25^+^Foxp3^+^) percentages of spleens were analyzed by flow cytometry. (e) Th17 (CD3e^+^CD4^+^IL‐17A^+^) percentages of the spleens were analyzed by flow cytometry. *n* = 3–7 per group. Statistical analysis was performed via one‐way ANOVA. *p < 0.05, **p < 0.01, ***p < 0.001

Commensal microbes can alter metabolites thereby modifying host‐generated signaling molecules.^26^ Untargeted metabolome profiles were used to evaluate whether the therapeutic effect of EcN‐Sj16 on colitis is related to its effect on colonic metabolism. We found the metabolites were significantly different between EcN‐Sj16 treatment and EcN treatment (Figure 5(a) and Table S2). Interestingly, among these differential metabolites, the level retinoic acid was significantly higher in EcN‐Sj16 treatment group than in EcN treatment group (Figure 5(a) and Table S2). In addition, we examined the level of the retinoic acid in serum and colon tissue by ELISA. As shown in Figure 5(b), retinoic acid in serum and colon tissue was increased by treating with EcN‐Sj16 compared with EcN treatment. Furthermore, we found that retinoic acid receptor α (RARA) was upregulated after treatment with EcN‐Sj16 (Figure 5(c)).

Retinoic acid inhibits the differentiation of naïve T cells to Th17 cells and increases the differentiation of naïve T cells towards Tregs.^27^, ^28^ RARA is critical for the normal differentiation and functions of Tregs.^29^ Improving Treg/Th17 balance contributed to the re‐establishment of gut immune homeostasis and reduced IBD.^30^ Therefore, we assessed the changes in Treg and Th17 levels in spleens by flow cytometry. We observed that the percentages of CD3e^+^CD4^+^CD25^+^Foxp3^+^ (Treg) cells of EcN‐Sj16‐treated group were increased compared with EcN‐treated group (*p* < 0.01) (Figure 5(d)). In addition, we noted that the percentages of CD3e^+^CD4^+^IL‐17A^+^ (Th17) cells of EcN‐Sj16‐treated group were significantly lower (*p* < 0.001) than EcN‐treated group (Figure 5(e)).

### 2.7 EcN‐Sj16 attenuated clinical activity of colitis mice via Ruminococcaceae/butyrate/retinoic acid axis

**FIGURE 6:**
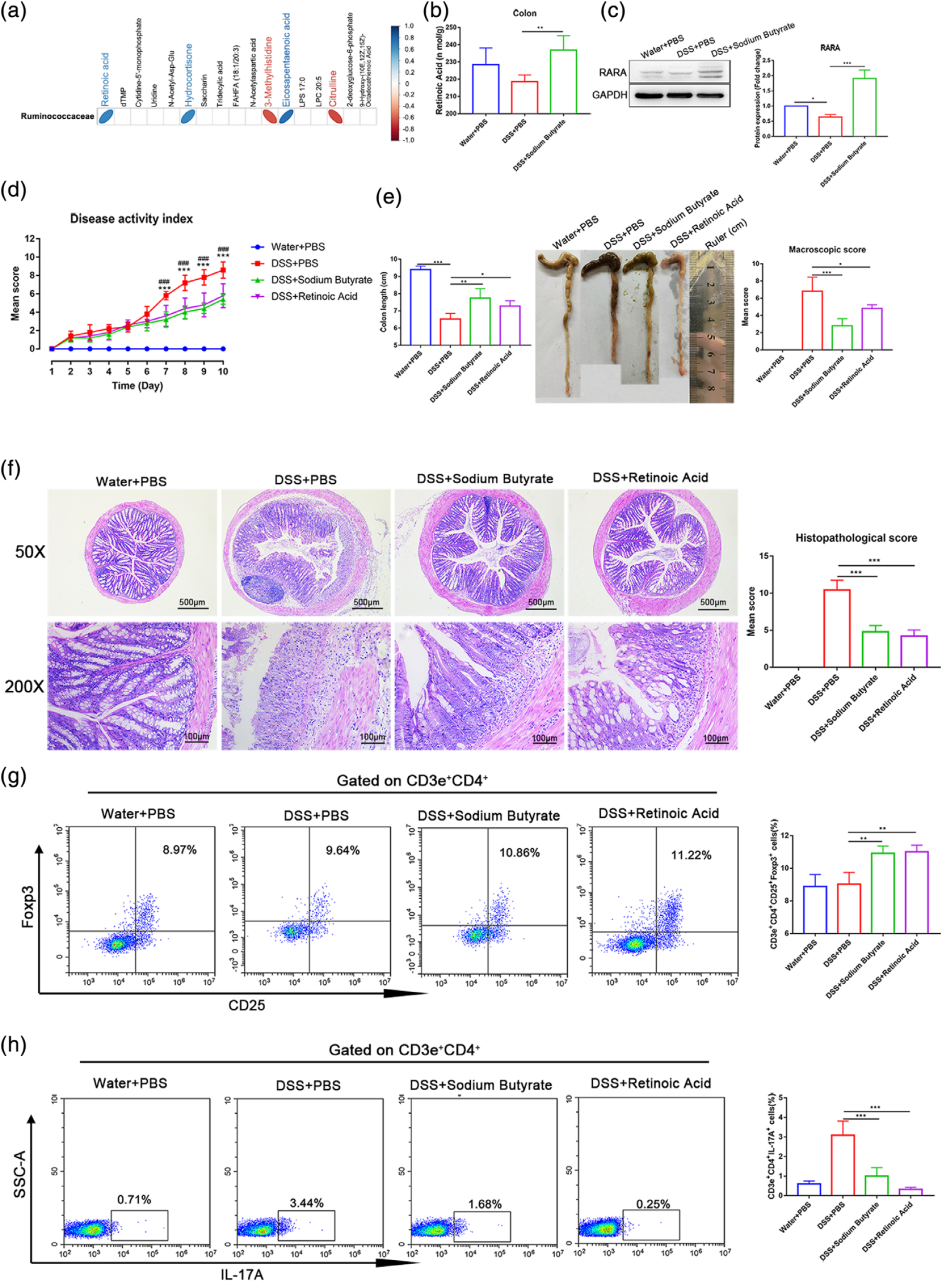
EcN‐Sj16 attenuated clinical activity of colitis mice via Ruminococcaceae/butyrate/retinoic acid axis. (a) The correlations between colon metabolite modules and the gut microbiota, the color represents positive (blue) or negative (red) correlations. (b) The level of the retinoic acid in colon tissue was examined by ELISA. (c) RARA was analyzed by western blotting. (d) Changes in DAI (*: DSS + sodium butyrate vs. DSS + PBS; #: DSS + retinoic acid vs. DSS + PBS). (e) Colon lengths and mean macroscopic scores of colons were measured and recorded. (f) Histopathological changes in the colons were examined via H&E staining and histopathological scores were determined. (g) Treg (CD3e^+^CD4^+^CD25^+^Foxp3^+^) percentages of spleens were analyzed by flow cytometry. (h) Th17 (CD3e^+^CD4^+^IL‐17A^+^) percentages of the spleens were analyzed by flow cytometry. *n* = 5 per group. Statistical analysis was performed via one‐way ANOVA. *p < 0.05, **p < 0.01, ***p < 0.001, ###*p* < 0.001

We subsequently assessed the correlation between the gut microbiota and colon metabolites in EcN‐Sj16‐treated colitis mice. We found retinoic acid was positively correlated with butyrate producer‐Ruminococcaceae (Figure 6(a)). In addition, the level of the retinoic acid in colon tissue of colitis mice was increased by treating with sodium butyrate (Figure 6(b)). Moreover, consistent with retinoic acid, RARA was upregulated after treatment with sodium butyrate (Figure 6(c)).

Next, to verify whether EcN‐Sj16 attenuated clinical activity of colitis mice via Ruminococcaceae/butyrate/retinoic acid axis, colitis mice were treated with sodium butyrate and retinoic acid. As expected, the results showed that the DAIs of sodium butyrate‐treated colitis mice and retinoic acid‐treated colitis mice were lower than that of the PBS‐treated colitis mice (Figure 6(d)). The colon lengths of sodium butyrate‐treated colitis mice and retinoic acid‐treated colitis mice were longer than those of the PBS‐treated, and the mean macroscopic colon scores of colitis mice were significantly decreased after treatment with sodium butyrate and treatment with retinoic acid (Figure 6(e)). Both sodium butyrate and retinoic acid significantly reduced the colonic architecture disruption and histological scores compared with that of the PBS treatment (Figure 6(f)). We further assessed the changes in Treg and Th17 subset levels in spleens by flow cytometry. The percentages of CD3e^+^CD4^+^CD25^+^Foxp3^+^ (Treg) cells of sodium butyrate‐treated group and retinoic acid‐treated group were significantly higher than PBS‐treated group (Figure 6(g)), and the percentages of CD3e^+^CD4^+^IL‐17A^+^ (Th17) cells of sodium butyrate‐treated group and retinoic acid‐treated group were lower than PBS‐treated group (Figure 6(h)). Interestingly, we observed that the percentages of CD3e^+^CD4^+^IFN‐γ^+^ (Th1) cells were significantly decreased after treatment with sodium butyrate and treatment with retinoic acid (Figure S3).

Thus, we conclude that EcN‐Sj16 attenuated clinical activity of colitis mice via Ruminococcaceae/ butyrate/retinoic acid axis.

### 2.8 Retinoic acid inhibitor inhibited the therapeutic effects of EcN‐Sj16 in DSS‐induced colitis mice

**FIGURE 7:**
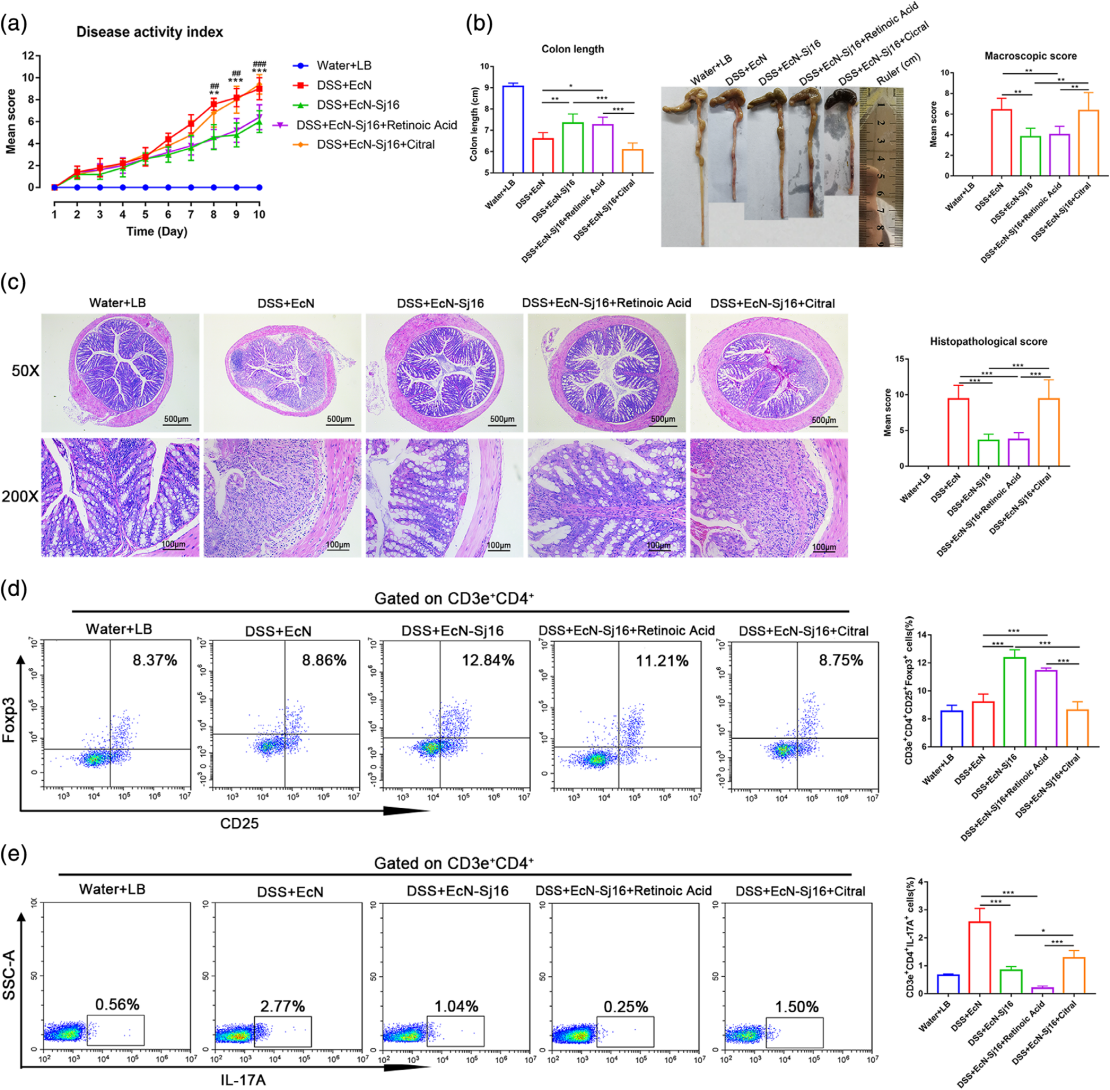
Retinoic acid inhibitor inhibited the therapeutic effects of EcN‐Sj16 in DSS‐induced colitis mice. (a) Changes in DAI (*: DSS + EcN‐Sj16 vs. DSS + EcN‐Sj16 + Citral; #: DSS + EcN‐Sj16 + Retinoic Acid vs. DSS + EcN‐Sj16 + Citral). (b) Colon lengths and mean macroscopic scores of colons were measured and recorded. (c) Histopathological changes in the colons were examined by H&E staining and histopathological scores were determined. (d) Treg (CD3e^+^CD4^+^CD25^+^Foxp3^+^) percentages of spleens were analyzed by flow cytometry. (e) Th17 (CD3e^+^CD4^+^IL‐17A^+^) percentages of the spleens were analyzed by flow cytometry. *n* = 5 per group. Statistical analysis was performed via one‐way ANOVA. *p < 0.05, **p < 0.01, ***p < 0.001, ###*p* < 0.0.001

To further evaluate retinoic acid plays a key role in the therapeutic effects of EcN‐Sj16, we used citral (a retinoic acid synthesis inhibitor) to inhibit retinoic acid. As expected, DAIs of DSS + EcN‐Sj16 + citral group were markedly high than DSS + EcN‐Sj16 group and DSS + EcN‐Sj16 + retinoic acid group (Figure 7(a)). Additionally, retinoic acid inhibition resulted in a significant reduction in colon length in the DSS + EcN‐Sj16 + citral group compared with DSS + EcN‐Sj16 group and DSS + EcN‐Sj16 + retinoic acid group (Figure 7(b)). Mean colon macroscopic scores were significantly increased in the DSS + EcN‐Sj16 + citral group compared with DSS + EcN‐Sj16 group and DSS + EcN‐Sj16 + retinoic acid group (Figure 7(b)). Moreover, DSS + EcN‐Sj16 + citral group exhibited significant colonic architecture disruption and histological scores increased compared with DSS + EcN‐Sj16 group and DSS +EcN‐Sj16 + retinoic acid group (Figure 7(c)).

As shown in Figure 7(d), retinoic acid inhibition resulted in a significant reduction of the percentages of CD3e^+^CD4^+^CD25^+^Foxp3^+^ (Treg) cells in the DSS + EcN‐Sj16 + citral group compared with DSS + EcN‐Sj16 group and DSS + EcN‐Sj16 + retinoic acid group (Figure 7(d)), and the percentage of CD3e^+^CD4^+^IL‐17A^+^ (Th17) cells of DSS + EcN‐Sj16 + citral group were higher than DSS + EcN‐Sj16 group and DSS + EcN‐Sj16 + retinoic acid group (Figure 7(e)). In addition, the percentages of CD3e^+^CD4^+^IFN‐γ^+^ (Th1) cells were increased in DSS + EcN‐Sj16 + citral group compared with DSS + EcN‐Sj16 group and DSS + EcN‐Sj16 + retinoic acid group (Figure S4). Together, our findings showed that retinoic acid plays a key role in the therapeutic effects of EcN‐Sj16 in DSS‐induced colitis mice.

## 3 DISCUSSION

In this study, we adopt a new strategy to attenuate colitis using the *Escherichia coli* Nissle 1917 probiotic strain to express a *schistosome* immunoregulatory protein (Sj16) in the gastrointestinal tract. We confirmed that EcN‐Sj16 was highly efficient in ameliorating the clinical activity of DSS‐induced colitis. EcN‐Sj16 attenuated clinical activity of colitis mice via Ruminococcaceae/butyrate/retinoic acid axis, and retinoic acid inhibitor inhibited the therapeutic effects of EcN‐Sj16 in DSS‐induced colitis mice.

Engineered bacteria administration may help clinically manage IBD and studies have been proposed to explain its benefits.^14^, ^31^ Human trials have used bacterial mixtures, such as VSL#3, or single probiotics such as EcN.^32^ One study showed that EcN had a similar effect to mesalazine in maintaining remission among UC patients.^13^ EcN can colonize the human intestines, with no virulence factors, as a safe and effective probiotic for treating colitis.^33^, ^34^ Our group showed that Sj16 has anti‐inflammatory effects in vitro and in vivo. In vitro, Sj16 downregulates lipopolysaccharide‐induced proinflammatory cytokine production and upregulates immunoregulatory cytokines in dendritic cells and macrophages.^35^, ^36^ In vivo, Sj16 was reported to reduce arthritis severity in a rat model and suppress thioglycolate‐mediated leukocyte recruitment to mouse peritoneal cavities.^37^ Furthermore, Sj16 protects against DSS‐induced colitis.^12^ Therefore, we genetically engineered the EcN to secrete Sj16 (EcN‐Sj16) and found that EcN‐Sj16 has therapeutic effects in DSS‐induced colitis mice. In addition, our results showed that the therapeutic effects of EcN‐Sj16 were significantly superior to that of EcN. Therefore, we believe that Sj16 plays a major role in EcN‐Sj16 ameliorates DSS‐induced colitis.

Previous studies have shown reduced biodiversity in the gut microbiota compositions of IBD patients, characterized by reductions in beneficial bacteria and increased pathogens or pathobionts.^19^, ^20^, ^38^ EcN‐Sj16 survive in the gut and has direct contact with the gut microbiota. Therefore, we hypothesized that the therapeutic effect of EcN‐Sj16 on DSS‐induced colitis was closely related to its influence on the gut microbiota composition. After EcN‐Sj16 treatment, both the α‐diversity and β‐diversity were significantly increased. EcN and EcN‐Sj16 similarly influenced the gut microbiota diversity of the DSS‐induced colitis mice. However, EcN‐Sj16 more strongly attenuated the clinical activity of DSS‐induced colitis mice than did EcN. We further found Ruminococcaceae family was significantly increased in EcN‐Sj16 treatment group than EcN treatment group. Ruminococcaceae is a major butyrate producer and is dramatically reduced in IBD patients.^22^ Fecal metabolomic analyses verified that butyrate was significantly associated with clinical remission in IBD patients following anti‐TNF therapy.^21^ Butyrate can act directly on intestinal macrophages to reduce inflammatory cytokine production and can ameliorate colitis by maintaining Treg/Th17 balance,^39^ inhibiting histone deacetylase 1 and maintaining epithelial barrier integrity.^40^ We found sodium butyrate in serum and colon tissue was increased by treating with EcN‐Sj16. This suggests that the therapeutic effect of EcN‐Sj16 on DSS‐induced colitis is closely related to Ruminococcaceae.

Commensal microbes can alter metabolites thereby modifying host‐generated signaling molecules.^26^ Study showed that a large number of serum metabolites increase due to commensal microbes.^41^ It has been established that changes in microbiota can affect the metabolic pathways and immune system.^42^, ^43^, ^44^, ^45^ We found the metabolites were significantly different in colons of colitis mice between EcN‐Sj16 and EcN treatment. Interestingly, the level of retinoic acid and RARA were significantly higher in EcN‐Sj16 treatment group than in EcN treatment group. Studies have indicated that Retinoic acid inhibits the differentiation of naïve T cells to Th17 cells and increases the differentiation of naïve T cells towards Tregs,^27^ and Retinoic acid can reduce the severity of the IBD.^46^ Our previous study revealed that Sj16 has protective effects on DSS‐induced colitis by inhibiting the PPAR‐α signaling pathway.^12^ Study has shown that retinoic acid has opposite regulating effect on PPAR‐α gene expression.^47^ Therefore, there is a correlation between the protective effects on DSS‐induced by inhibiting the PPAR‐α signaling pathway and ameliorates colitis via increasing retinoic acid. All‐trans retinoid acid can prevent Tregs from converting to Th1/Th17 cells and sustains Tregs suppressive function in inflammatory environments.^48^ RARA is important for the differentiation and functions of Tregs and Th1 cells.^29^ We observed that the percentages of Treg cells of EcN‐Sj16‐treated group were increased compared with EcN‐treated group. In addition, the percentages of Th17 cells of EcN‐Sj16‐treated group were significantly lower than EcN‐treated group.

Imbalance between Th17 and Treg cell triggers the progression of colitis.^30^ Improving Treg/Th17 balance contributed to the re‐establishment of gut immune homeostasis and reduced IBD.^30^ During gut microbiota dysbiosis, due to the excessive growth of pathogenic bacteria and reduced gut microbiota diversity, causing the Treg/Th17 balance disturbed and tend towards Th17 cells.^49^ Gut microbiota diversity and microbiota‐derived metabolites altering the Treg/Th17 balance play an important role in the inflammatory responses of IBD.^49^ Our results showed that retinoic acid was positively correlated with Ruminococcaceae. In addition, the level of the retinoic acid and RARA of colitis mice was increased by treating with sodium butyrate. Treated with retinoic acid and sodium butyrate attenuated clinical activity of colitis mice. The percentages of Treg cells in the spleens were increased and Th17 cells were decreased after treatment with EcN‐Sj16, sodium butyrate, and retinoic acid. Th1 cells profoundly accelerate the progression of IBD.^50^, ^51^ Interestingly, the percentages of Th1 cells were significantly decreased after treatment with sodium butyrate and retinoic acid. Thus, we conclude that EcN‐Sj16 attenuated clinical activity of colitis mice via Ruminococcaceae/butyrate/retinoic acid axis. And the therapeutic effects of EcN‐Sj16 in DSS‐induced colitis mice were inhibited by retinoic acid inhibition. In addition, the effects of EcN‐Sj16 in up‐regulated Treg cells and down‐regulated Th1 and Th17 cells were inhibited by retinoic acid inhibition.

Our work provides evidence for the utility of EcN‐Sj16 as a novel engineered probiotic for the treatment of IBD. Compared with Sj16 alone, sodium butyrate, and retinoic acid, EcN‐Sj16 protection should be long‐lasting because EcN‐Sj16 can colonize and replicate in the host. Although the efficient survival period in mice was only 4 days, colonization may be more effective in humans because EcN is a human strain rather than rodent strain. Like commercial EcN preparations (e.g., Mutaflor), EcN‐Sj16 should be able to be composed of lyophilized bacteria in an enteric capsule, which can be taken orally and stored indefinitely at room temperature. Finally, in addition to being effective in colitis, EcN‐Sj16 may be effective against other autoimmune diseases and thus requires further study.

## 4 MATERIALS AND METHODS

Detailed procedures are provided in Supporting Information.

### 4.1 Mice

Six‐week‐old male BALB/c mice (specific pathogen‐free, weighing 18–20 g) were purchased from the Guangdong Medical Laboratory Animal Center. The Medical Research Ethics Committee of Sun Yat‐sen University approved all animal experiments, which conformed to the Chinese National Institute of Health Guide for their Care. The mice were group‐housed in ventilated cages in a temperature‐controlled room (25°C) and fed standard mouse chow.

### 4.2 Expression and detection of Sj16 in EcN

To determine the protein expression, the transformants were grown overnight at 37°C in LB containing 100 μg/mL ampicillin. Cultures were centrifuged at 8000 × g for 10 min at 4°C, and equal volumes of the secreted proteins in the culture supernatant were concentrated using Amicon Ultra‐4 Centrifugal Filter Units (Millipore, Germany). Proteins in the supernatant and precipitate were separated by 10% SDS‐PAGE and transferred to a PVDF membrane (Millipore, Germany). Membranes were blocked in 5% milk and then incubated with mouse anti‐Sj16 antibody, followed by anti‐mouse IgG conjugated to horse‐radish peroxidase (Abcam, UK). Purified Sj16 was used as a positive control.

### 4.3 DSS‐induced colitis and treatment

To induce acute DSS colitis, drinking water with 2.5% (wt/vol) DSS (36–50 kDa, MP Biomedicals) was administered to the mice for 10 days (Days 1–10). Mice were gavaged with either LB, EcN or EcN‐Sj16 (1 × 10^9^ bacteria in 200 μL of LB) on Days 0, 4, and 8. The water + LB and DSS + LB groups were gavaged with 200 μL of LB. Sodium butyrate (50 μmol) (MedChemExpress), retinoic acid (400 μg) (MedChemExpress), citral (400 μg) (MedChemExpress), or vehicle were treated to mice from Days 1–9 via intraperitoneal injection. Fresh fecal samples were collected daily in the morning and assayed for EcN and EcN‐Sj16 strains by plating on agar plates containing ampicillin.

### 4.4 Clinical disease scoring

During treatment, changes in body weight and occurrences of diarrhea and bleeding were recorded. Fecal blood was identified using the Hemoccult assay kit (Nanjing Jiancheng Bio‐engineering Institute, China). Body weight changes were calculated relative to Day 1 body weight. Clinical disease scores (DAI) were evaluated based on weight loss, stool type, and bleeding as previously described.^12^

### 4.5 Macroscopic assessment and histologic evaluation

On Day 10, the mice were sacrificed, and their colon lengths were measured. An independent observer blinded to the treatment status assessed the macroscopic scores of the colons. Parameters of macroscopic scores include hyperemia, wall thickening, ulceration, inflammation extension, and damage as our previously described.^52^ The colons were fixed in 4% paraformaldehyde, routinely processed, and embedded in paraffin. Paraffin‐embedded colon sections were prepared and stained with hematoxylin and eosin (H&E). Histopathological scores were examined in a blinded fashion as previously described.^12^ Histopathological scores of the colonic lesions were determined based on inflammation extent, neutrophil and lymphohistiocyte infiltration, crypt damage extent, crypt abscess formation, submucosal edema, goblet cell loss, and reactive epithelial hyperplasia.

### 4.6 ELISA

Sodium butyrate and retinoic acid in serum and colon tissue were examined by ELISA kits, according to the manufacturer's instructions (JINGMEI, China).

### 4.7 Statistical analysis

Results are expressed as means ± SD values. Multiple comparisons were analyzed via one‐way analysis of variance (ANOVA) and the Wilcoxon rank‐sum test using GraphPad Prism software, version 7.0 (GraphPad Software, San Diego, CA). *p* < 0.05 was considered statistically significant.

## AUTHOR CONTRIBUTIONS

**Lifu Wang:** Investigation; methodology; writing‐original draft; writing‐review & editing. **Yao Liao:** Data curation; investigation; methodology. **Ruibing Yang:** Data curation; investigation; methodology. **Zifeng Zhu:** Data curation; methodology. **Lichao Zhang:** Data curation; methodology. **Zhongdao Wu:** Methodology; project administration; supervision; validation. **Xi Sun:** Formal analysis; funding acquisition; project administration; writing‐original draft.

## CONFLICT OF INTEREST

The authors declare that there is no conflict of interest that could be perceived as prejudicing the impartiality of the research reported.

### PEER REVIEW

The peer review history for this article is available at https://publons.com/publon/10.1002/btm2.10219.

## Supporting information

**Appendix** S1: Supporting Information

## Data Availability

Data for generating the figures could be obtained with reasonable request to Prof. Xi Sun.
