# Supplementary material for: An engineered probiotic secreting Sj16 ameliorates colitis via Ruminococcaceae/butyrate/retinoic acid axis
Source: Bioeng Transl Med. 2021 Apr 2;6(3):e10219. doi: 10.1002/btm2.10219 (PMC8459592; doi:10.1002/btm2.10219)
Supplement: Supplementary file 1 — Appendix S1: Supporting Information [file BTM2-6-e10219-s001.docx]

**Supporting Information**

**An engineered probiotic secreting Sj16 ameliorates colitis via Ruminococcaceae/butyrate/retinoic acid axis**

Lifu Wang^1,2,3^, Yao Liao^1,2,3^, Ruibing Yang^1,2,3^, Zifeng Zhu^1,2,3^, Lichao Zhang^1,2,3^, Zhongdao Wu^1,2,3^, Xi Sun ^1,2,3^*

^1^Department of Parasitology of Zhongshan School of Medicine, Sun Yat-sen University, Guangzhou 510080, China.

^2^Key Laboratory of Tropical Disease Control, Ministry of Education, Sun Yat-sen University, Guangzhou 510080, China.

^3^Provincial Engineering Technology Research Center for Biological Vector Control, Guangzhou 510080, China.

*Corresponding author:

Xi Sun, Department of Parasitology of Zhongshan School of Medicine, Sun Yat-sen University, Guangzhou, 510080, China. Email address: [sunxi2@mail.sysu.edu.cn](mailto:sunxi2@mail.sysu.edu.cn)

**Supplementary materials and methods**

**1. Bacterial strains and culture conditions**

EcN and *E. coli* DH5a were purchased from The Query Network for Microbial Species of China. *E. coli* J96 was purchased from the American Type Culture Collection. pGEX-4T-1 plasmids were preserved in our laboratory. *E. coli* DH5a was used for DNA cloning and plasmid amplification. *E. coli* J96 (uropathogenic *E. coli*; UPEC) was used to clone the HlyA secretion system. EcN, *E. coli* DH5a and *E. coli* J96 were cultured in Luria-Bertani broth (LB) or on agar plates at 37°C. Ampicillin (100 μg/ml) was added to the media when applicable.

**2. Plasmid construction**

The α-hemolysin secretion system was from *E. coli* J96. This system expressed and secreted the Sj16 protein, and the plasmid was transferred into EcN (EcN-Sj16). The cDNA library of adult *Schistosoma japonicum* (Chinese mainland strain) was used as the PCR template. The upstream primer was 5'-CGGAATTCTTGATCACAGCTACAACGTTAG-3' (the underlined portion was the *EcoRI* restriction site). The downstream primer was 5'-CGCGTCGACCTAAGACGATTCATAT-3' (the underlined portion was the site digested by *Sal I*). The prokaryotic expression plasmid, pGEX-4T-1, and the purified Sj16 were digested and ligated to construct the pGEX-4T-1-Sj16 vector. Using UPEC J96 genomic DNA as a template, *AsBD* fragments (including the carboxyl terminal signal sequences of the *HlyA*, *HlyB* and *HlyD* gene sequences) were amplified using primers (Forward: 5′- TGATGGAATATGAATCGTCTGAGCTCCATTAGCCTATGGAAGTCAGGGTAA-3′; Reverse 5′- AGTCAGTCACGATGCGGCCGCTTAAGACTTAACGCTCATGTAAACTTTCTG-3′). The pGEX-4T-1-Sj16 vector was connected with the *AsBD* fragment (pGEX-4T-1-Sj16-AsBD). The characteristic of the plasmid is that the Sj16 gene is fused with the secretory signal sequence *hlyAs* in the same frame and under the control of the promoter of pGEX-4T-1. The pGEX-4T-1-Sj16-AsBD plasmid was transferred into the EcN. All constructs were verified via PCR and DNA sequencing. For GFP-tagged bacteria, GFP-AsBD gene sequence was amplified using primers (Forward: 5′- TGATGGAATATGAATCGTCTATGCGTAAAGGCGAAGAGC-3′; Reverse 5′- AGTCAGTCACGATGCGGCCGCTTAAGACTTAACGCTCATGTAAACTTTCTG-3′). The pGEX-4T-1-Sj16 vector was then connected with the GFP-AsBD fragment (pGEX-4T-1-Sj16-GFP-AsBD), and the plasmid was transferred into EcN.

**3. DNA extraction and 16S rRNA gene sequencing**

Total genomic DNA was extracted from feces in the colon, jejunum and ilea (day 10) using the CTAB/SDS method. DNA concentration and purity were monitored and diluted to 1 ng/µl using sterile water. 16S rRNA genes of distinct regions (V3–V4/16S) were amplified. Loading buffer (containing SYB green) was mixed with PCR products and electrophoresed on agarose gel for DNA detection. PCR products were mixed at equidensity ratios and purified using the Qiagen Gel Extraction Kit (Qiagen, Germany). Sequencing libraries were generated using the TruSeq® DNA PCR-Free Sample Preparation Kit (Illumina, USA) and assessed on the Qubit@ 2.0 Fluorometer (Thermo Scientific) and Agilent Bioanalyzer 2100 system. The library was sequenced on an Illumina NovaSeq platform and 250-bp paired-end reads were generated.

**4. 16S rRNA gene sequence analysis**

Paired-end reads were merged. Raw tags were quality filtered under specific filtering conditions to obtain high-quality clean tags. Chimera sequences were removed, and effective tags were obtained. Sequences were analyzed using Uparse software (Uparse v7.0.1001, <http://drive5.com/uparse/>), and those with ≥97% similarity were assigned to the same OTUs. For representative sequences, the Silva Database (<http://www.arb-silva.de/>)was used based on a Mothur algorithm to annotate taxonomic information. Multiple sequence alignment was conducted using MUSCLE software (version 3.8.31, <http://www.drive5.com/muscle/>) {Edgar, 2004 #1014} to study the phylogenetic relationships of the OTUs and differences in the dominant species. OTU abundances were normalized using a standard of sequence number corresponding to the sample with the fewest sequences. Alpha-diversity indices were calculated using QIIME (version 1.7.0) and displayed using R (version 2.15.3). Beta diversity was calculated in QIIME (version 1.9.1). Cluster analysis was performed using principal coordinate analysis (PCoA) and UPGMA clustering.

**5. Untargeted metabolomics study**

Colon tissues were individually grounded with liquid nitrogen. After centrifuged, the supernatant was injected into the LC-MS/MS system analysis. UHPLC-MS/MS analyses were performed by a Vanquish UHPLC system (Thermo Fisher, Germany) coupled with an Orbitrap Q ExactiveTM HF-X mass spectrometer (Thermo Fisher，Germany) in Novogene Co., Ltd. (Beijing, China). The raw data files from UHPLC-MS/MS were processed by the Compound Discoverer 3.1 (CD3.1, Thermo Fisher) to perform peak alignment, peak picking, and quantitation for each metabolite. Peak intensities were normalized to the total spectral intensity, and the normalized data was used to predict the molecular formula. And then peaks were matched with the mzCloud (<https://www.mzcloud.org/)，mzVault> and MassList database.

Metabolites were annotated by the KEGG database ( <https://www.genome.jp/> kegg/pathway.html), HMDB database(https://hmdb.ca/ metabolites) and LIPID Maps database (http://www.lipidmaps.org/). Principal components analysis (PCA) and Partial least squares discriminant analysis (PLS-DA) were performed at metaX. Univariate analysis (t-test) was used to calculate the statistical significance (P-value). We applied volcano plots to filter metabolites of interest. For clustering heat maps, the data were normalized using z-scores of the intensity areas of differential metabolites and were ploted by Pheatmap package in R language.

**6. Multi-omic analyses**

Multi-omic analyse was performed between the significantly different flora obtained from 16S rRNA and the significantly different metabolites obtained from metabonomics analysis. The value range of correlation coefficient is (-1 to 1). When the correlation coefficient is less than 0, there is a negative correlation. When the correlation coefficient is greater than 0, it is positively correlated. When equal to 0, there is zero correlation.

**7. Western blotting**

Colon tissue were homogenized with RIPA lysis buffe (Thermo Fisher Scientific, USA). Lysates were subjected to 10% sodium dodecyl-polyacrylamide gel electrophoresis and transferred to a polyvinylidene fluoride blotting membrane (GE Healthcare Life Sciences, UK). The membranes were then immunoblotted with RARA antibody (10331-1-AP, Proteintech, China). The membranes were visualized by ECL Western blotting detection system (Amersham, USA). Image J software was used to determine intensity of pixels.

**8. Flow cytometry**

Single-cell suspensions were prepared. Treg, Th17 and Th1 subsets were analyzed by flow cytometry. For Treg subsets, the cells were stained with fluorochrome-conjugated CD3e (557596, BD Biosciences, USA), CD4 (553052, BD Biosciences, USA), and CD25 (47-0257-41, eBiosciences, USA) antibodies; the cells were then stained with Foxp3 (12-4776-41, eBiosciences, USA) antibodies after fixation and permeabilization. For Th17and Th1 subsets, cells were cultured with 50 ng/ml phorbol 12-myristate 13-acetate (PMA), 500 ng/mL ionomycin, and 10 μg/mL brefeldin A (Sigma-Aldrich, USA) for 6 h. Then, cells were stained for CD3e (557596, BD Biosciences, USA) and CD4 (553052, BD Biosciences, USA) antibodies. In addition, cells were stained with IL-17A (563354, BD Biosciences, USA) and IFN-γ (557724, BD Biosciences, USA) antibodies. Images were acquired on a CytoFLEX S flow cytometer (Beckman Coulter, USA).

**Supplementary Figures**

**
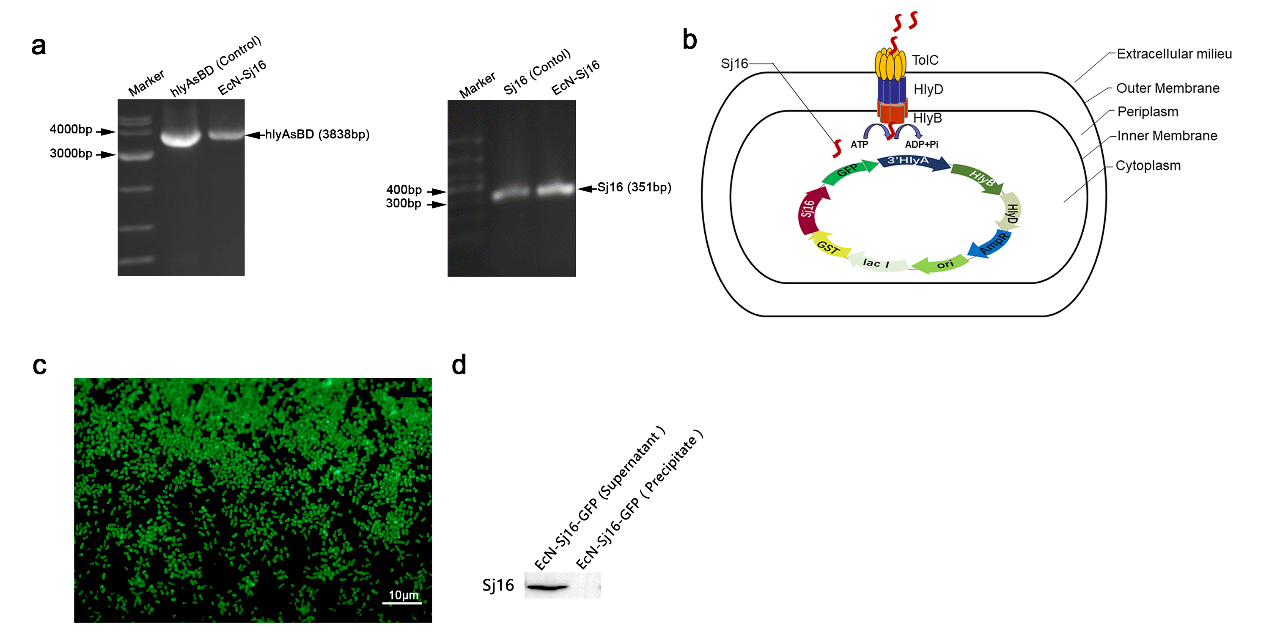
**

**FIGURE S1** (a) EcN-Sj16 was identified by PCR. (b) Schematic representation of the EcN-Sj16-GFP constructs. The Hly type I secretion system consisted of the inner-membrane components, HlyB and HlyD, and the outer-membrane component, TolC. The inner and outer membranes form a membrane pore. The HlyB-HlyD complex recognizes the C-terminal portion of HlyA to guide direct export of HlyA-fusion Sj16-GFP from the cytoplasm into the extracellular environment, bypassing the periplasmic space. (c) EcN-Sj16-GFP with an expression vector expressing green fluorescent protein (GFP), HlyA, Sj16, HlyB, and HlyD. (d) Sj16 of EcN-Sj16-GFP expressed in the supernatant and precipitate was analyzed by western blotting.


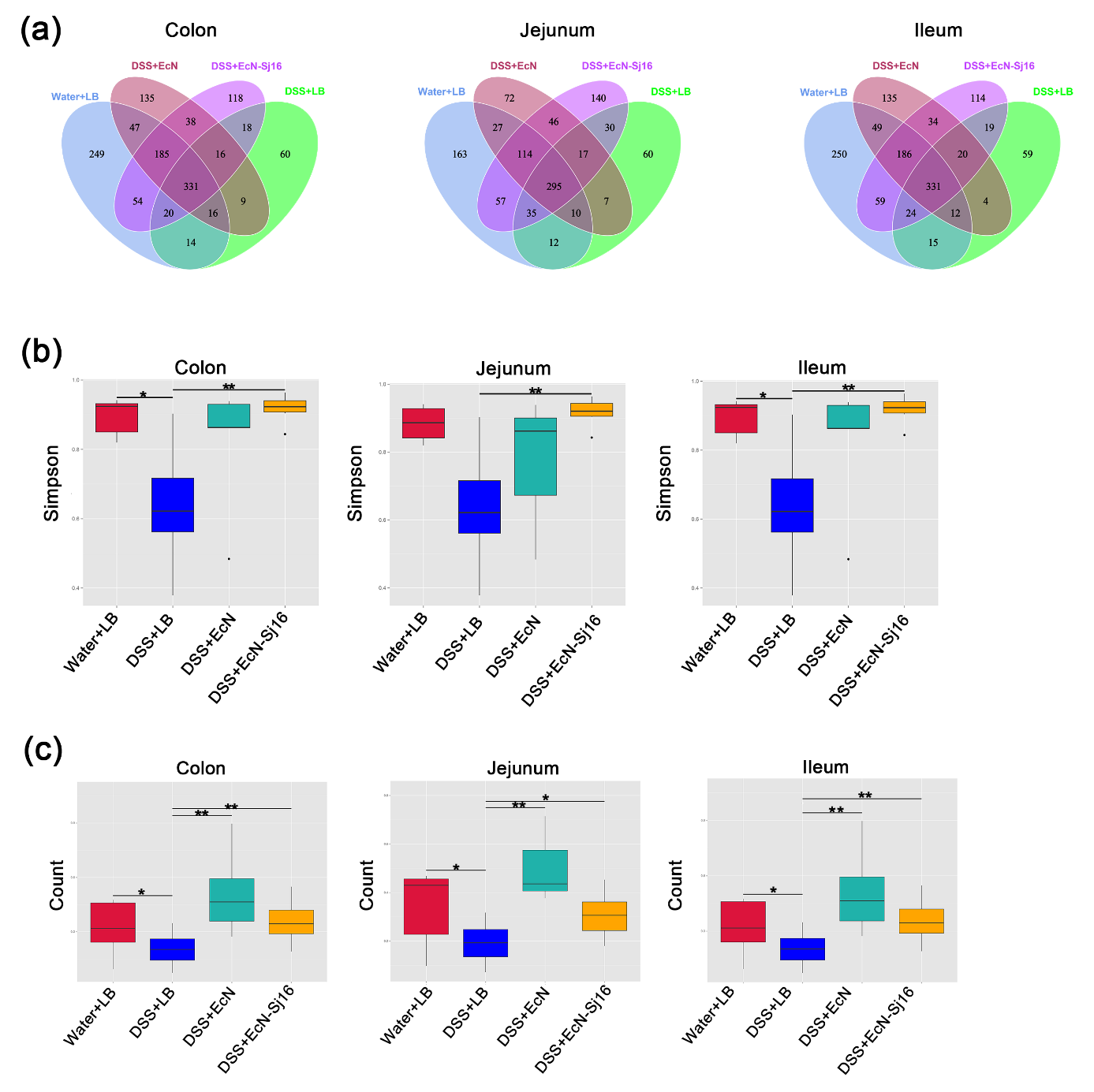


**FIGURE S2** EcN-Sj16 rescued the gut microbial compositions of DSS-induced colitis mice. (a) Venn diagram showing the overlapping OTUs in each group. (b) α-diversity indices were estimated using the Simpson diversity index. (c) Interindividual dissimilarities were assessed using weighted UniFrac distance-based β-diversity indices. n=3–7 per group. Significant differences in α-diversity and β-diversity were assessed via the Wilcoxon rank-sum test. **P* <0.05, ***P* <0.01.

**
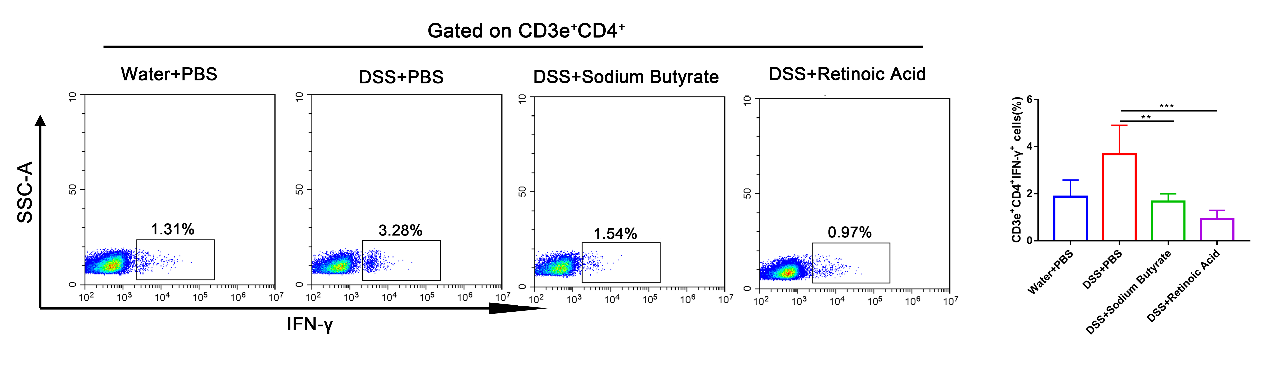
**

**FIGURE S3** After treated with sodium butyrate and retinoic acid, CD3e^+^CD4^+^IFN-γ^+^ (Th1) percentages of the spleens were analyzed by flow cytometry. n=5 per group. Statistical analysis was performed via one-way ANOVA. ***P* <0.01, ****P* <0.001.


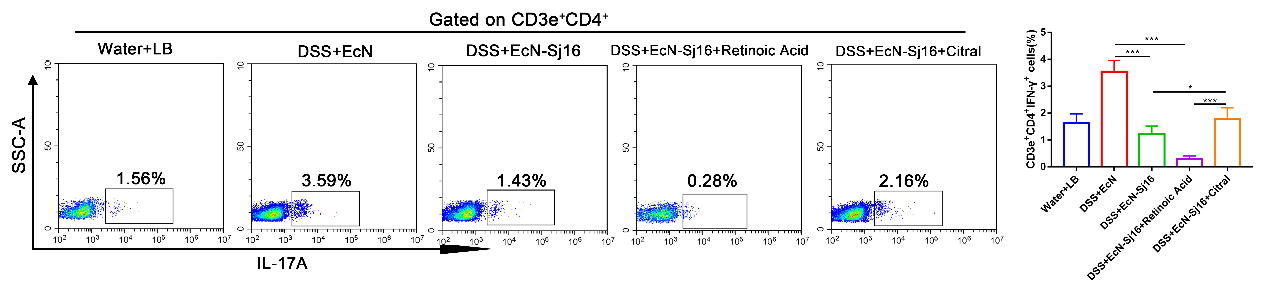


**FIGURE S4** Citral was used to inhibit retinoic acid, and CD3e^+^CD4^+^IFN-γ^+^ (Th1) percentages of the spleens were analyzed by flow cytometry. n=5 per group. Statistical analysis was performed via one-way ANOVA. **P* <0.05, ****P* <0.001.
